# Supplementary material for: Ovarian torsion: A retrospective case series at a tertiary care center emergency department
Source: PLoS One. 2024 Mar 6;19(3):e0297690. doi: 10.1371/journal.pone.0297690 (PMC10917280; doi:10.1371/journal.pone.0297690)
Supplement: Attachment — Submitted filename: Response to Reviewers.docx [file pone.0297690.s002.docx]

**Rebuttal Letter that responds to each point raised by the academic editor and reviewers**

Dear Drs. Fabio Vasconcellos, Emily Chenette and Iain Hrynaszkiewicz,

Thank you very much for your positive response and for considering our manuscript for publication at your esteemed journal. We are submitting a revised version of the manuscript after addressing the minor comments as per the academic editor and reviewers.

1. Please ensure that your manuscript meets PLOS ONE's style requirements, including those for file naming. The PLOS ONE style templates can be found at 
https://journals.plos.org/plosone/s/file?id=wjVg/PLOSOne_formatting_sample_main_body.pdf and 
https://journals.plos.org/plosone/s/file?id=ba62/PLOSOne_formatting_sample_title_authors_affiliations.pdf

Thank you for your comment. We ensured that the manuscript meets the PLOS ONE’s style requirements.

2. Note from Emily Chenette, Editor in Chief of PLOS ONE, and Iain Hrynaszkiewicz, Director of Open Research Solutions at PLOS:

Did you know that depositing data in a repository is associated with up to a 25% citation advantage (https://doi.org/10.1371/journal.pone.0230416)? If you’ve not already done so, consider depositing your raw data in a repository to ensure your work is read, appreciated and cited by the largest possible audience. You’ll also earn an Accessible Data icon on your published paper if you deposit your data in any participating repository (https://plos.org/open-science/open-data/#accessible-data).

Thank you for your note. We appreciate you informing us about depositing the raw data in a repository.

3. In your Data Availability statement, you have not specified where the minimal data set underlying the results described in your manuscript can be found. PLOS defines a study's minimal data set as the underlying data used to reach the conclusions drawn in the manuscript and any additional data required to replicate the reported study findings in their entirety. All PLOS journals require that the minimal data set be made fully available. For more information about our data policy, please see http://journals.plos.org/plosone/s/data-availability.

"Upon re-submitting your revised manuscript, please upload your study’s minimal underlying data set as either Supporting Information files or to a stable, public repository and include the relevant URLs, DOIs, or accession numbers within your revised cover letter. For a list of acceptable repositories, please see http://journals.plos.org/plosone/s/data-availability#loc-recommended-repositories. Any potentially identifying patient information must be fully anonymized.

Important: If there are ethical or legal restrictions to sharing your data publicly, please explain these restrictions in detail. Please see our guidelines for more information on what we consider unacceptable restrictions to publicly sharing data: http://journals.plos.org/plosone/s/data-availability#loc-unacceptable-data-access-restrictions. Note that it is not acceptable for the authors to be the sole named individuals responsible for ensuring data access.

We will update your Data Availability statement to reflect the information you provide in your cover letter.

Thank you for your comment. All our data is fully available without any restrictions. We totally agree of the importance of sharing our data used to reach the conclusions drawn and to replicate the study findings in their entirety. We uploaded the study’s minimal underlying data set as a supporting information file.

4. Please review your reference list to ensure that it is complete and correct. If you have cited papers that have been retracted, please include the rationale for doing so in the manuscript text, or remove these references and replace them with relevant current references. Any changes to the reference list should be mentioned in the rebuttal letter that accompanies your revised manuscript. If you need to cite a retracted article, indicate the article’s retracted status in the References list and also include a citation and full reference for the retraction notice.

Thank you for your comment. We have reviewed the reference list and ensured that it is complete and correct. We have added 1 additional reference in the discussion section and in the reference list to highlight the histopathologic findings noted in our study as compared to the literature.

20.	Vijayalakshmi K. Clinico-Pathological Profile of Adnexal Torsion Cases: A Retrospective Analysis from A Tertiary Care Teaching Hospital. J Clin Diagn Res [Internet]. 2014 [cited 2023 Dec 22]; Available from: <http://jcdr.net/article_fulltext.asp?issn=0973-709x&year=2014&volume=8&issue=6&page=OC04&issn=0973-709x&id=4456>

**Comments to the Author**

1. Is the manuscript technically sound, and do the data support the conclusions?

The manuscript must describe a technically sound piece of scientific research with data that supports the conclusions. Experiments must have been conducted rigorously, with appropriate controls, replication, and sample sizes. The conclusions must be drawn appropriately based on the data presented.

Reviewer #1: Yes

Reviewer #2: Yes

Thank you for your comment.

2. Has the statistical analysis been performed appropriately and rigorously?

Reviewer #1: Yes

Reviewer #2: Yes

Thank you for your comment.

3. Have the authors made all data underlying the findings in their manuscript fully available?

The [PLOS Data policy](http://www.plosone.org/static/policies.action#sharing) requires authors to make all data underlying the findings described in their manuscript fully available without restriction, with rare exception (please refer to the Data Availability Statement in the manuscript PDF file). The data should be provided as part of the manuscript or its supporting information, or deposited to a public repository. For example, in addition to summary statistics, the data points behind means, medians and variance measures should be available. If there are restrictions on publicly sharing data—e.g. participant privacy or use of data from a third party—those must be specified.

Reviewer #1: Yes

Reviewer #2: Yes

Thank you for your comment. All data underlying the findings described in the manuscript is fully available without restrictions. We uploaded the study’s minimal underlying data set as a supporting information file.

4. Is the manuscript presented in an intelligible fashion and written in standard English?

PLOS ONE does not copyedit accepted manuscripts, so the language in submitted articles must be clear, correct, and unambiguous. Any typographical or grammatical errors should be corrected at revision, so please note any specific errors here.

Reviewer #1: Yes

Reviewer #2: Yes

Thank you for your comment.

5. Review Comments to the Author

Please use the space provided to explain your answers to the questions above. You may also include additional comments for the author, including concerns about dual publication, research ethics, or publication ethics. (Please upload your review as an attachment if it exceeds 20,000 characters)

Reviewer #1: The topic is important and not rare at all. Although the final number of cases was twenty cases only but I know it is not ease to find a large number of cases with evident torsion especially in one hospital.

Thank you for your comment. Indeed, while the overall number of cases is not large, this incidence in one setting was and we highlighted this in our discussion section.

Reviewer #2: Dear author, the study is good. Kindly upload the histopathology report of the operated case. Was Doppler done in all cases along with USG. In how many cases the whirlpool sign was positive.

Thank you for your constructive suggestions. We uploaded the histopathology results of the operated cases in table 1, in a new column under the heading: “Histopathology report”. A total of 13 cases have a histopathology result. 7 cases do not have any histopathology results (marked as N/A in the table) given that either no specimen was taken during surgery or because of missing data. We have updated these results in the manuscript. We also noted that the most common histopathology finding was mature cystic teratoma. We discussed these findings in our discussion section.

Moreover, while reviewing the histopathology reports we have identified 5 additional cases of cystectomies, that were not previously noted in the US reports/operative findings. We have added these cases and modified these findings and percentages in the entire manuscript.

While all of the cases who underwent pelvic ultrasonography had a color doppler examination done, only 13 of the reported interpretations commented on the doppler findings. We have documented these doppler findings in table 1 under the heading: “Doppler ultrasonography findings” and subheading “Doppler” when available. In addition, even though the whirlpool sign has a high sensitivity and specificity in the diagnosis of ovarian torsion, it was not documented as a terminology in any of the ultrasonographic reports by the radiology team at our tertiary care center.

6. PLOS authors have the option to publish the peer review history of their article ([what does this mean?](https://journals.plos.org/plosone/s/editorial-and-peer-review-process#loc-peer-review-history)). If published, this will include your full peer review and any attached files.


If you choose “no”, your identity will remain anonymous but your review may still be made public.


**Do you want your identity to be public for this peer review?** For information about this choice, including consent withdrawal, please see our [Privacy Policy](https://www.plos.org/privacy-policy).

Reviewer #1: No

Reviewer #2: **Yes:** Prof Dr Varsha Deshmukh

Thank you for your comment.
